# Measurement of Scleral Thickness in Humans Using Anterior Segment Optical Coherent Tomography

**DOI:** 10.1371/journal.pone.0132902

**Published:** 2015-07-28

**Authors:** Hetal D. Buckhurst, Bernard Gilmartin, Robert P. Cubbidge, Nicola S Logan

**Affiliations:** 1 Plymouth University, School of Health Professions, Peninsula Allied Health Centre, Derriford Road, Plymouth, United Kingdom; 2 Aston University, School of Life & Health Sciences, Birmingham, United Kingdom; Charité University Medicine Berlin, GERMANY

## Abstract

Anterior segment optical coherent tomography (AS-OCT, *Visante*; Zeiss) is used to examine meridional variation in anterior scleral thickness (AST) and its association with refractive error, ethnicity and gender. Scleral cross-sections of 74 individuals (28 males; 46 females; aged between 18-40 years (27.7±5.3)) were sampled twice in random order in 8 meridians: [superior (S), inferior (I), nasal (N), temporal (T), superior-temporal (ST), superior-nasal (SN), inferior-temporal (IT) and inferior-nasal (IN)]. AST was measured in 1mm anterior-to-posterior increments (designated the A-P distance) from the scleral spur (SS) over a 6mm distance. Axial length and refractive error were measured with a Zeiss *IOLMaster* biometer and an open-view binocular Shin-Nippon autorefractor. Intra- and inter-observer variability of AST was assessed for each of the 8 meridians. Mixed repeated measures ANOVAs tested meridional and A-P distance differences in AST with refractive error, gender and ethnicity. Only right eye data were analysed. AST (mean±SD) across all meridians and A-P distances was 725±46μm. Meridian SN was the thinnest (662±57μm) and I the thickest (806±60μm). Significant differences were found between all meridians (p<0.001), except S:ST, IT:IN, IT:N and IN:N. Significant differences between A-P distances were found except between SS and 6 mm and between 2 and 4 mm. AST measurements at 1mm (682±48 μm) were the thinnest and at 6mm (818±49 μm) the thickest (p<0.001); a significant interaction occurred between meridians and A-P distances (p<0.001). AST was significantly greater (p<0.001) in male subjects but no significant differences were found between refractive error or ethnicity. Significant variations in AST occur with regard to meridian and distance from the SS and may have utility in selecting optimum sites for pharmaceutical or surgical intervention.

## Introduction

Information on the properties of scleral tissue is apposite to our understanding of the aetiology of myopia [1], glaucoma [2], age-related macular degeneration (AMD) [3], surgical procedures [4–5] and the pharmacokinetics of drug transmission to the posterior globe [6–7].

A specific and accessible property of sclera tissue, anterior scleral thickness (AST), has previously been determined *in vivo* for specific meridians using ultrasound biometry (UBM) [8–10], ultrasound pachymetry [11] and anterior segment optical coherent tomography [12–13]. Relatively low-resolution T-1 weighted 2-D MRI *in vivo* measurements of posterior chamber scleral thickness in humans have also been reported [8, 14] and, more recently, high-field 3-D microMRI *in vitro* measurements in 11 (7 normal, 4 glaucomatous) enucleated human eyes [15]. In the latter study, substantial differences in thickness were observed between the inferior (greatest thickness), nasal, temporal and superior (least thickness) anterior ocular quadrants [15].

Much of the previous literature concerning intra-individual and inter-individual variation in AST has concerned individuals with myopia [9, 16] or glaucoma [10, 13]. In their UBM study, Oliveira *et al*., [9] found thickness along the anterior temporal meridian to correlate positively with increasing axial length (AL) and myopic refractive error but this was not corroborated by Yoo *et al*., [13]. Central corneal thickness (CCT) has been considered an independent risk factor in glaucoma [17] and a positive correlation between CCT and AST has been reported by several groups but the relationship appears to be dependent on the category of glaucoma [10, 13]. Several studies have found greater scleral thickness in males [10, 18], whilst other have failed to confirm these observations [13, 19]. Furthermore, data on differences in scleral thickness between ethnic groups appears to be confined to Caucasian and non-Caucasian individuals, the former having thinner temporal sclera thickness [9].

To address further intra-meridian and intra-subject variability the present study uses anterior segment optical coherent tomography (AS-OCT) to measure *in vivo* a series of scleral thickness measurements (AST) for each meridian within the region between the limbus and ora serrata (6-7mm from the limbus) in 74 adult male and female subjects of mixed ethnicity and refractive error and who are free of ocular pathology.

## Method

### Subjects

Subjects were recruited from the staff and student population at Aston University. Eligibility to take part in the study was confirmed after subjects completed a screening questionnaire. As ocular biomechanics have previously been shown to be affected by various ocular diseases and conditions, the exclusion criteria included previous history of ocular surgery, trauma or pathology, ocular medication and astigmatism >1.75 D. Furthermore, individuals suffering from connective tissue related disorders were also excluded due to their known affect on collagen composition and hence scleral biomechanics [20]. The majority of subjects were either students or staff from the Optometry School and hence had undergone regular and detailed ocular screening ensuring awareness of any existing ocular pathology.

Seventy-four individuals (148 eyes; 28 males and 46 females; aged between 18–40 years (27.7 ± 5.3) of British White (BW, n = 48), British South Asian (BSA, n = 23) and other ethnic (n = 3) origin gave written informed consent prior to participating in the study. Subjects were categorized as non-myopic [mean spherical error (MSE (D)) ≥ -0.50] n = 37 MSE mean ± SD (0.49 ± 1.08), range (-0.50 to +4.38)) or myopic [MSE (D) <-0.50] n = 37 MSE mean ± SD (-4.22 ± 3.17) range (-0.51 to -10.56). Ethical approval was obtained from Aston University Ethics Committee and the study was performed according to the tenets of the Declaration of Helsinki.

### Biometric measurements

Cycloplegia was induced using 1 drop of tropicamide HCI 1% (*Minims*, Bausch & Lomb, Kingston-Upon-Thames, UK). An objective measure of the refractive error and central corneal curvature was determined with a binocular open view autorefractor/keratometer (Shin-Nippon SRW-5000, Ryusyo Industrial Co. Ltd, Osaka, Japan). Five measurements were taken, averaged and converted to MSE (sphere power + 0.5 x cylinder power). AL and anterior chamber depth (ACD) measurements were taken with the commercially available *IOLMaster* (Carl Zeiss Meditec, Inc., Dublin, California, USA). Five separate measurements were averaged for AL, whereas three separate measurements were averaged for central corneal curvature (mm), and a single capture shot automatically generated and averaged five measurements of ACD. Corneal pachymetry measurements were obtained with the global pachymetry scan protocol on the AS-OCT (*Visante*; Carl Zeiss Meditec). The *Visante* system automatically processes 16 line scans and represents a map of the pachymetry values. The average reading for the central 2 mm was used for analysis.

### Scleral image acquisition

Scleral cross-sectional images up to approximately 6 mm posterior from the scleral spur (SS) were acquired using the enhanced high-resolution corneal scan mode of the *Visante* AS-OCT. The *Visante* is a temporal domain OCT that has an axial resolution of 18 μm and transverse resolution of 60 μm and offers a 16 mm x 6 mm scanning field (www.meditec.zeiss.com). The high-resolution images are generated from 512 A-scans in 250 ms (*Visante* manual). High resolution transverse and axial (transverse x axial) 10 x 3 mm cross-sectional images with a 512 x 1024 pixel resolution (Version 2) were acquired in random order in 8 different meridians: superior (S), inferior (I), nasal (N), temporal (T), superior-temporal (ST), superior-nasal (SN), inferior-temporal (IT) and inferior nasal (IN). To access the scleral sections, subjects were asked to fixate in eight different directions external to the instrument and the imaging plane angle was altered to match the meridian being scanned. The scanning acquisition plane was set to 180, 45, 90 and 135 degrees to capture the 8 meridians of gaze. The *Visante* allows manipulation of the chin/headrest and image plane to ensure the anatomical features of interest were being imaged. The examiner observed a real-time image of the subject’s eye on the video monitor, thus allowing easier manipulation and precision of alignment. In the vertical and oblique gazes the lids were gently retracted by the patient to expose as much of the sclera as possible. To avoid any order effects, the sequence of imaging of each meridian was randomised. Randomisation of the order for imaging the eight meridians was generated in Microsoft *Excel* (Microsoft Corporation, Redmond, Washington, USA).

### Scleral image analysis

To minimise and partially correct the effects of optical distortion produced by variation in refractive indices of the anterior segment tissues [21], the *Visante* software applies in ‘analysis mode’ a refractive index of 1.00 (air) to all structures exterior to the anterior corneal surface, 1.388 (cornea) for all structures within the corneal section and 1.343 (aqueous) to all structures posterior to the back surface of the cornea [22]. For expediency a refractive index of 1.388 was assumed for the sclera and the seven *Visante* on-screen measurement calipers were used to measure anatomical features with resolution increments of 10 μm [13]. All meridians were measured manually in random order by the same examiner (HB) using the protocol described below.

**Fig 1:**
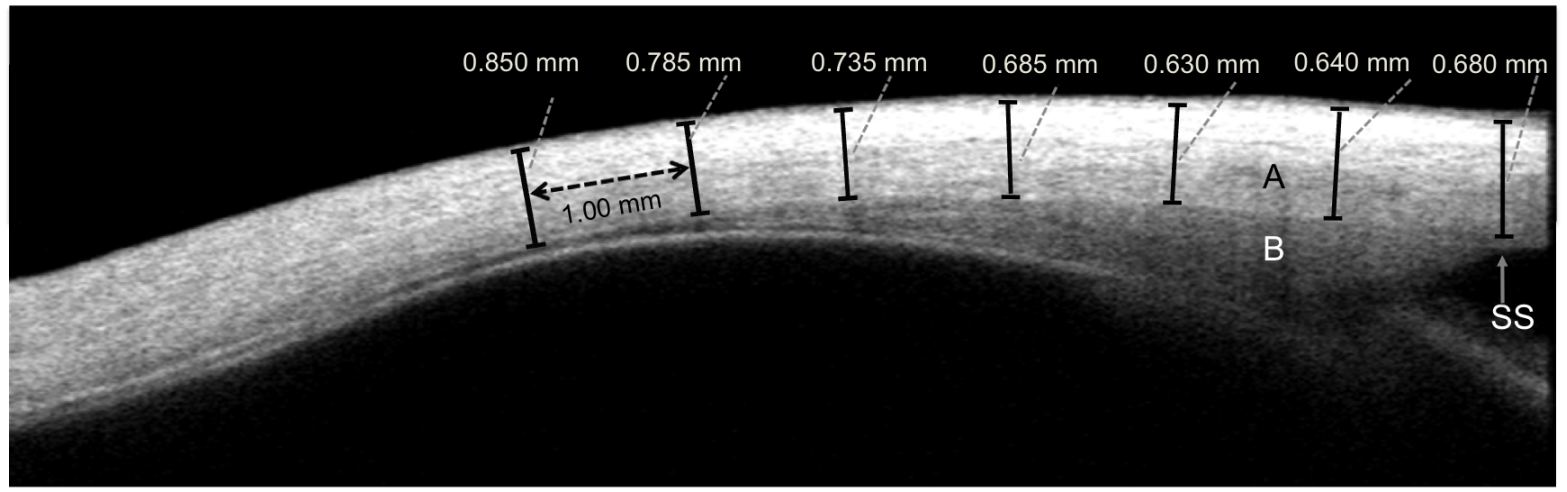
OCT image of the temporal sclera (A). Ciliary muscle (B); Scleral spur (SS). Calipers (in black) shown for scleral thickness measurements for 6 mm from the SS at 1 mm intervals (in dotted black).

The location of the apex of the SS was determined by identifying manually the point where there is a slight protrusion of sclera into the anterior chamber and where the ciliary muscle fibres initially meet the scleral tissue at the irido-scleral junction (Fig 1) [23]. This apex was used as a measurement fulcrum. The caliper orientation and separation were then manipulated to be, respectively, approximately tangential to and contiguous with the scleral interfaces delineated by differential image reflectivity, the separation thus providing a value for scleral thickness. The process was repeated for successive anterior-to-posterior (A-P) increments of 1mm out to 6 mm from the SS.

To assess intraobserver variability in AST, 7 separate cross-sectional images were captured and assessed for each of the 8 meridians for one subject in random order by one investigator (HB) without knowledge of which meridian was being evaluated. In order to determine interobserver variability in AST, 2 separate cross-sectional images were captured and assessed for each of the 8 meridians for 11 subjects in random order independently by two investigators (HB and ED) without knowledge of which meridian was being evaluated. To ensure masking, the second examiner would load the image on the AS-OCT screen and cover up all details that would identify which meridian was being assessed. Due to the method in which the cross-sectional images were captured it was not possible for the examiners to identify which meridian was being assessed. Despite being masked to the meridian being assessed the examiner was not blind to the measurement values as manual calipers available via the OCT software were used to determine the AST values.

### Statistical analysis

Only the right eye (RE) AST data were analysed. Statistical evaluation was performed using *SPSS* version 15 for Windows (SPSS Inc, Chicago, IL) and Microsoft *Excel* (Microsoft Corporation, Redmond, Washington, USA). To assess the intraobserver variability for measures of AST, the coefficient of variation (CoV; CoV% = (standard deviation/mean) * 100) was calculated for each meridian and distance from the SS. Interobserver variability was evaluated by calculating intraclass correlation coefficient (ICC; two-way mixed single measures (consistency agreement); ICC (consistency, k (number of raters) = subject variability / (subject variability + measurement error/k)) for each meridian and distance in *SPSS*.

Two-way repeated and three-way mixed repeated measures ANOVAs were performed with meridian (8 levels) and distance (mm from SS, 7 levels) as within-subject factors and between-subject factors as: refractive error (MSE, non-myopic ((MSE ≥ -0.50) n = 37 MSE (D) mean ± SD (0.49 ± 1.08), range (-0.50 to +4.38)) and myopic ((MSE <-0.50) n = 37 MSE (D) mean ± SD (-4.22 ± 3.17) range (-0.51 to -10.56)), 2 levels), low, intermediate and high AL (mm, 1. (21.5>- ≤ 23.5) n = 26, 2. (23.5 >- ≤25.5) n = 33, 3. (>25.5, 3 levels) n = 14), ethnicity (BW n = 48, BSA n = 23, 2 levels) and gender (male n = 28 and female n = 46, 2 levels). Pearson’s product-moment correlation coefficient was used to test for relationships between AST, CCT, MSE and ocular biometric parameters. Student’s *t test* was used to test differences in ocular biometry between gender and ethnicity. For all statistical tests a p-value of <0.05 was taken as the criterion for statistical significance.

### Intraobserver variability

Good repeatability was found for all 8 meridians and A-P distances with CoV being generally of the order of 4%; greatest CoV was observed for the IN (5.82%) meridian and at the SS (5.04%) and lowest variability along the temporal (2.82%) meridian and at the 3mm (3.75%) distance.

### Interobserver variability

ICC values for the interobserver variability demonstrated high levels of repeatability between examiners for the AST measurements. Similar levels of repeatability were found for all meridians (average ICC 0.930; lowest ICC N:0.899 and highest ICC IN:0.961) and distances (average ICC 0.929; lowest ICC at SS: 0.882 and highest ICC at 2mm: 0.955). For comparison of inter-observer repeatability, 95% limits of confidence were also assessed [24]. The mean difference between the repeated measurements (i.e. average AST measurements for each meridian by the two examiners) represents the bias of the method. The mean and standard deviation (SD) of differences were used to calculate 95% limits of agreement (LoA: mean ± [1.96 * SD]) Table 1. The LoA represents the expected differences between repeated measurements.

**Table 1:** Interobserver repeatability of AST measurements for the 8 meridians (superior-nasal (SN), superior (S), superior-temporal (ST), temporal (T), inferior-temporal (IT), inferior (I), inferior-nasal (IN) and nasal (N)).

Meridian	Bias	SD of Differences	95% LoA
SN	6.3	18.9	-30.7, +43.4
S	7.0	23.2	-38.8, +52.1
ST	3.7	15.0	-25.7, +33.1
T	6.0	11.2	-16.1, +27.8
IT	-4.7	30.6	-64.6, +55.2
I	-8.0	36.4	-78.9, +63.4
IN	5.0	17.2	-28.8, +38.8
N	7.0	22.3	-36.8, +50.7

## Results

### Biometric parameters

Independent Student’s *t test* analyses revealed no significant difference in ocular biometric parameters (i.e. MSE, AL, CCT, ACD and corneal curvature) for either gender or ethnicity.

### Variation in scleral thickness

#### Meridian and distance from sclera spur

**Fig 2:**
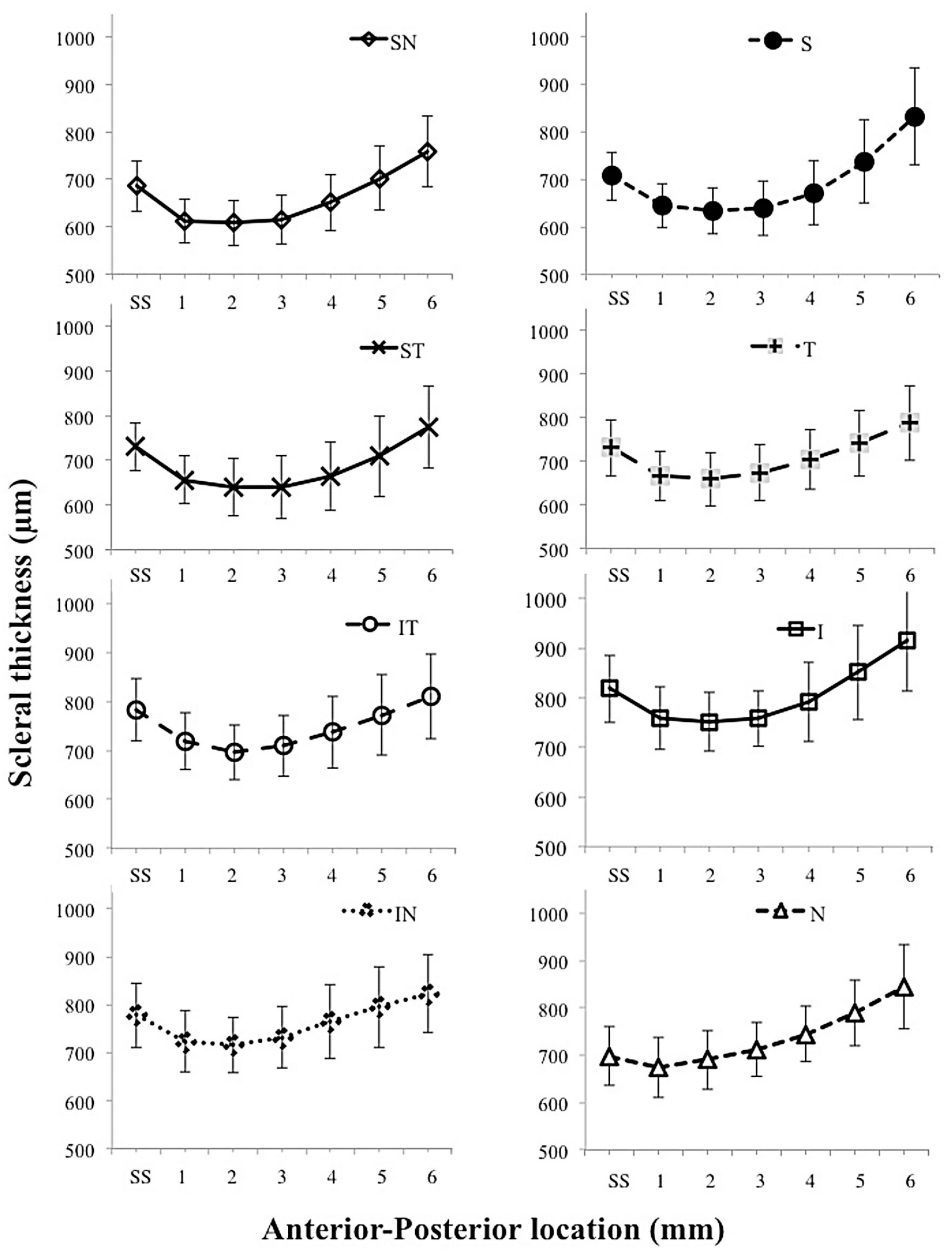
AST for each of the 8 meridians individually. Mean AST ± standard deviation for each meridian from SS and 1-6mm anterior-posterior distances from SS.

**Fig 3:**
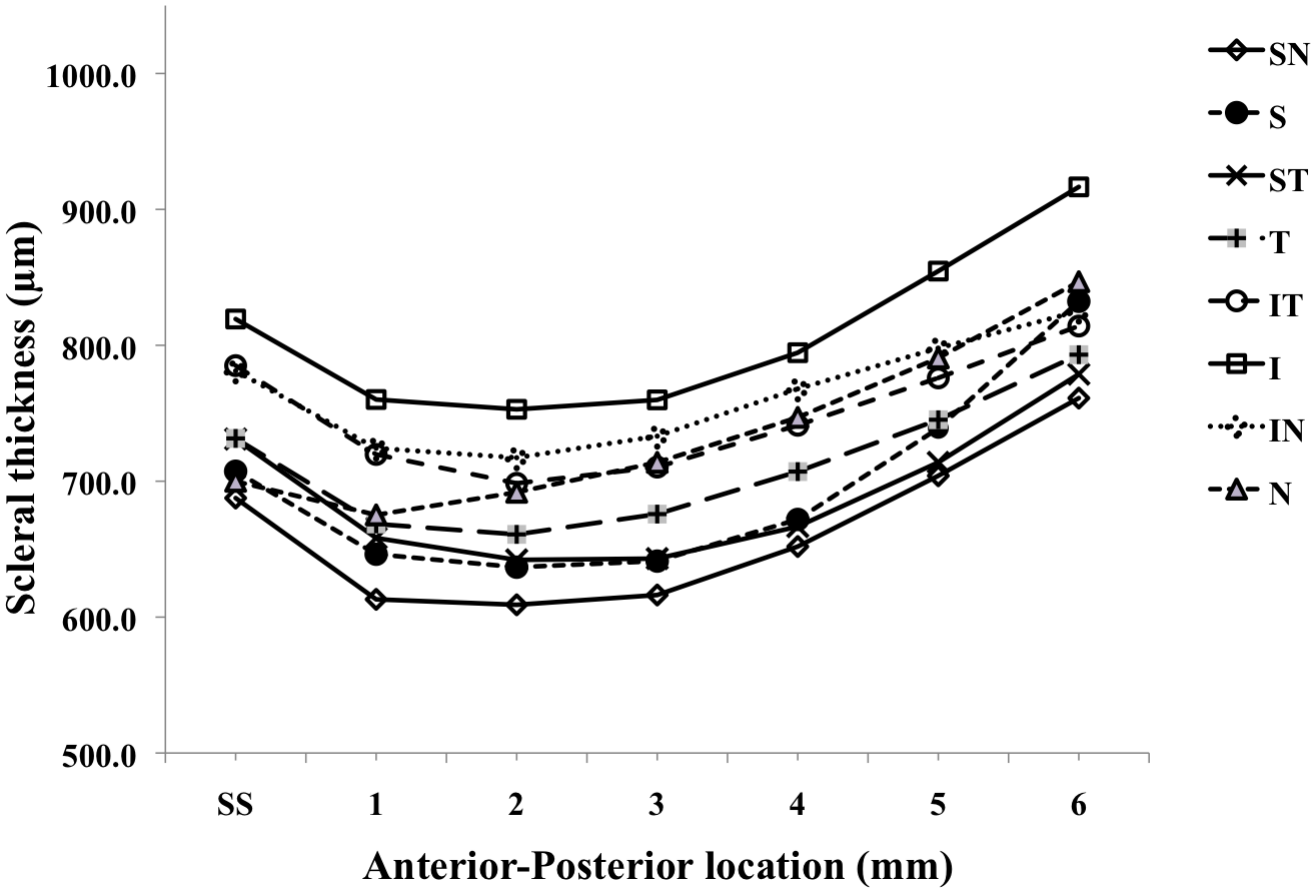
AST for all 8 meridians. Mean AST ± standard deviation at SS and 1-6mm anterior-posterior distances from SS.

A significant difference (p<0.001) in AST was observed for the different meridians; with a Bonferroni *post hoc* analysis demonstrating a significant difference between all meridians except between S:ST, IT:IN, IT:N and IN:N. The mean scleral thickness across all distances was maximum for the I meridian and minimum for the SN meridian (p<0.001) (Table 2). Similarly significant differences were identified for distance of measurement (p<0.001); with a Bonferroni *post hoc* analysis showing significant differences between all distances except between SS and 6 mm and between 2 and 4 mm. The general trend for all meridians indicates an initial decline in AST from the SS to the 2 mm increment and then a gradual increase from 3 mm onwards (Figs 2 and 3). Furthermore a significant interaction effect was found between meridians and distance of measurement (p<0.001), indicating that the AST at any particular distance (SS to 6 mm) would vary with the meridian being examined.

**Table 2:** RE AST for each meridian (superior-nasal (SN), superior (S), superior-temporal (ST), temporal (T), inferior-temporal (IT), inferior (I), inferior-nasal (IN) and nasal (N)) and distance (1–6 mm) from the SS (mean ± SD (μm)).

Meridians	AST at SS and distances from SS (mm)							
	SS	1	2	3	4	5	6	Average thickness
**SN**	686 ± 53	612 ± 47	608 ± 48	615± 51	651 ± 59	703 ± 67	760 ± 75	662 ± 57
**S**	706 ± 51	644 ± 46	634 ± 49	639 ± 57	671 ± 68	737 ± 87	831 ± 102	695 ± 71
**ST**	730 ± 53	656 ± 53	639 ± 65	640 ± 71	663 ± 76	709 ± 90	774 ± 92	687 ± 52
**T**	731 ± 64	666 ± 57	659 ± 61	673 ± 65	705 ± 68	741 ± 75	788 ± 86	709 ± 47
**IT**	783 ± 63	718 ± 58	696 ± 56	709 ± 62	737 ± 74	772 ± 83	810 ± 87	746 ± 42
**I**	818 ± 67	759 ± 63	751 ± 59	758 ± 56	792 ± 80	851 ± 95	915 ± 102	806 ± 60
**zIN**	778 ± 67	723 ± 63	716 ± 58	732 ± 64	765 ± 78	795 ± 84	823 ± 81	762 ± 40
**N**	698 ± 63	674 ± 63	690 ± 62	712 ± 57	745 ± 58	789 ± 69	845 ± 89	736 ± 61
**Average thickness**	741 ± 47	682 ± 48	674 ± 48	685 ± 51	716 ± 52	762 ± 50	818 ± 49	725 ± 46

#### Refractive error and axial length

Refractive error failed to show any significant effect on AST (p = 0.119). Similarly a non-significant effect (p = 0.907) was found for AL (mm) grouping.

#### Gender and ethnicity

A significant difference (p<0.001) in AST was found for gender with males (750±60) showing greater overall thickness than females (712±68); although the pattern of change in AST across the meridians (p = 0.054) and distance from SS (p = 0.086) was not significantly affected by gender. No significant effect was found for ethnicity (p = 0.190).

#### Scleral thickness and additional biometric parameters

Independent Student’s *t test* analyses revealed no significant difference in ocular biometric parameters (i.e. MSE, AL, CCT, ACD and corneal curvature) between gender and ethnicity. No significant correlations were observed between AST and AL or MSE. CCT correlated negatively (p<0.05) with AST along the SN (3–4 mm), S (4–6 mm), ST (4–6 mm), IT (4–5 mm) and I (5 mm) meridians.

## Discussion

The study is the first to report on the *in vivo* measurement of AST for different meridians in individuals without ocular pathology. Both intra- and inter-observer reliability of measurement of AST were found to be good and were able to demonstrate significant differences in AST for different meridians across a range of anterior-posterior distances from the scleral spur, notably that the inferior meridian has the thickest AST and the superior-nasal meridian the thinnest.

With reference to previous studies that have incorporated meridional measurements of AST, Norman *et al*., [15] used 3-dimensional microMRI to measure *in vitro* scleral thickness for circumferential slices of whole eyes of normal and glaucomatous patients. The slices were subdivided into nasal, temporal, superior and inferior meridians and mean sclera thickness plotted for each of 12 slices. Although the authors noted that significant intra-individual and inter-individual variation occurred, of interest is that, for the slices congruent with the measurement distances used to the present study, there was a clear differentiation in scleral thickness between meridians. The inferior meridian was found to be substantially thicker than the other three meridians such that respective meridians could be ranked in decreasing order of thickness as inferior, nasal, temporal and superior. Of note is that the ranking matched that for the equivalent meridians used in the present study (see Table 2): inferior 808μm, nasal 738μm, temporal 712μm, superior 696 μm and demonstrates that the *in vivo* data of the present study align well with the *post mortem* data presented by Norman *et al*., [15].

Elsheikh et al., [11] used ultrasound pachymetry on enucleated eyes to measure scleral thickness from the posterior retinal pole to the limbus for eight different meridians equivalent approximately to those used in the present study. Significant differences in scleral thickness were found between anterior, equatorial and posterior regions of the globe but the differences were independent of the meridian measured. It is unclear why meridional differences were not observed by Elsheikh et al., despite measures of anterior scleral thickness in the anterior segment region being similar (600–800 μm) to those of the present study. In comparison, using the same AS-OCT instrument as that employed in the present study Taban *et al*., [12] examined AST in patients implanted with fluocinolone acetonide steroid implants and observed non-uniform AST with significantly increased inferior-nasal thickness. Furthermore, a trend for decreasing scleral thickness from inferior-nasal followed by inferior-temporal, superior-temporal and superior-nasal was reported.

Our findings on regional variations in AST may have clinical relevance. Intravitreal injections are used for a variety of ophthalmological procedures and although current practice is for the injection to be applied through the inferior-temporal sclera (presumably due to ease of access) there appears to be no consensus on which location is optimum [25]. In a study investigating the effects of intravitreal injection site on vitreal reflux Turgut *et al*., [4] reported that injection through the inferior-temporal quadrant induced significantly less vitreal reflux when compared to the superior-temporal quadrant. With reference to the findings of the present study the thicker scleral profile of the inferior regions, particularly the inferior and inferior-nasal meridians, appear likely to minimise risks of post-operative complications. Intravitreal injections are known to cause transient increase in IOP, with peaks of 70 mmHg reported after injection [26]. It is unclear what factors contribute to this IOP increase, although measures of anterior scleral thickness have been found to show a positive correlation with the magnitude of IOP change [5].

*In vitro* studies have also shown that fluid flow and solute diffusion across the sclera is influenced by scleral thickness [27] and that the sclera is selectively permeable to different hydrophilic compounds of varying molecular size [6–7] although it is uncertain whether permeability varies with topographical location [28]. Table 2 and Fig 3 may therefore have utility in selecting optimum sites for intraocular drug absorption as they indicate that the superior-nasal meridian presents the thinnest AST and the inferior meridian the thickness AST at between 2 and 4mm from the limbus.

We were unable to demonstrate that AST was significantly affected by refractive error and axial length. The evidence for thinning of the anterior sclera with axial elongation is therefore equivocal compared with the well documented thinning of the posterior sclera that occurs in highly myopic eyes [29, 30]. Overall the mean AST was 6% thicker in males than females but AST followed similar patterns in both males and females with regard to respective meridians and distances from the sclera spur.

## References

[1] McBrien NA. Regulation of scleral metabolism in myopia and the role of transforming growth factor-beta. Exp Eye Res. 2013; 114:128–140. 10.1016/j.exer.2013.01.014 23399866

[2] Eilaghi A, Flanagan JG, Simmons CA. Ethier CR. Effects of scleral stiffness properties on optic nerve head biomechanics. Ann Biomed Eng. 2010; 38: 1586–1592. 10.1007/s10439-009-9879-7 20039133

[3] Pallikaris IG, Kymionis GD, Ginis HS, Kounis GA, Christodoulakis E, Tsilimbaris MK. Ocular rigidity in patients with age-related macular degeneration. Am J Ophthalmol. 2006; 141: 611–615. 16564793 10.1016/j.ajo.2005.11.010

[4] Turgut B, Demir T, Celiker U. The effects of injection site on the reflux following intravitreal injections. J Clin Med Res. 2009; 1: 280–284. 10.4021/jocmr2009.12.1280 22481990 PMC3311443

[5] Fuest M, Kotliar K, Walter P, Plange N. Monitoring intraocular pressure changes after intravitreal Ranibizumab injection using rebound tonometry. Ophthalmic Physiol Opt. 2014; 34: 438–444. 10.1111/opo.12134 24731161

[6] Lee SB, Geroski DH, Prausnitz MR, Edelhauser HF. Drug delivery through the sclera: effects of thickness, hydration, and sustained release systems. Exp Eye Res. 2004; 78: 599–607. 15106940 10.1016/s0014-4835(03)00211-2

[7] Olsen TW, Aaberg SY, Geroski DH, Edelhauser HF. Human sclera: thickness and surface area. Am J Ophthalmol. 1998; 125: 237–241. 9467451 10.1016/s0002-9394(99)80096-8

[8] Lam A, Sambursky RP, Maguire JI. Measurement of scleral thickness in uveal effusion syndrome. Am J Ophthalmol. 2005; 140: 329–331. 16086964 10.1016/j.ajo.2005.02.014

[9] Oliveira C, Tello C, Liebmann J, Ritch R. Central corneal thickness is not related to anterior scleral thickness or axial length. J Glaucoma. 2006; 15: 190–194. 16778639 10.1097/01.ijg.0000212220.42675.c5

[10] Mohamed-Noor J, Bochmann F, Siddiqui MAR, Atta HR, Leslie T, Maharajan P. et al Correlation between corneal and scleral thickness in glaucoma. J Glaucoma. 2009; 18: 32–36. 19142132 10.1097/IJG.0b013e31816b2fd1

[11] Elsheikh A, Geraghty B, Alhasso D, Knappett J, Campanelli M, Rama P. Regional variation in the biomechanical properties of the human sclera. Exp Eye Res. 2010; 90: 624–633. 10.1016/j.exer.2010.02.010 20219460

[12] Taban M, Lowder CY, Ventura AACM, Sharma S, Nutteret B, Hayden BC. et al Scleral Thickness following Fluocinolone Acetonide Implant (Retisert). Ocul Immunol Inflamm. 2010; 18: 255–263. 10.3109/09273941003658292 20482407

[13] Yoo C., Eom YS, Suh YW, Kim YY. Central Corneal Thickness and Anterior Scleral Thickness in Korean Patients With Open-angle Glaucoma: An Anterior Segment Optical Coherence Tomography Study. J Glaucoma. 2010; 20: 95–99. 10.1097/IJG.0b013e3181dde051 20577104

[14] Atchison DA, Pritchard N, Schmid KL, Scott DH, Jones CE, Pope JM. Shape of the retinal surface in emmetropia and myopia. Invest Ophthalmol Vis Sci. 2005; 46: 2698–2707. 16043841 10.1167/iovs.04-1506

[15] Norman RE, Flanagan JG, Rausch SMK, Sigal IA, Tertinegg I, Eilaghi A. et al Dimensions of the human sclera: Thickness measurement and regional changes with axial length. Exp Eye Res. 2009; 90: 277–284. 10.1016/j.exer.2009.11.001 19900442

[16] Schmid K., Li R., Edwards M. The expandability of the eye in childhood myopia. Curr Eye Res. 2003; 26, 65–71. 12815524 10.1076/ceyr.26.2.65.14513

[17] Dimasi DP, Burdon KP, Craig JE. The genetics of central corneal thickness. Br J Ophthalmol. 2010; 94: 971–976. 10.1136/bjo.2009.162735 19556215

[18] Watson PG, Young RD. Scleral structure, organisation and disease. A review. Exp Eye Res. 2004; 78: 609–623. 15106941 10.1016/s0014-4835(03)00212-4

[19] Vurgese S, Panda-Jonas S, Jonas JB. Scleral thickness in human eyes. PLOS ONE. 2012; 7: e29692 10.1371/journal.pone.0029692 22238635 PMC3253100

[20] Kaiser-Kupfer M.I., Podgor M.J., McCain L., Kupfer C., Shapiro J.R. Correlation of ocular rigidity and blue sclerae in osteogenesis imperfecta. Trans Ophthalmol Soc UK. 1985; 104, 191–195. 3857778

[21] Radhakrishnan S, Rollins AM, Roth JE, Yazdanfar S, Westphal V, Bardenstein DS. et al Real-time optical coherence tomography of the anterior segment at 1310 nm. Arch Ophthalmol. 2001; 119: 1179–1185. 11483086 10.1001/archopht.119.8.1179

[22] Lehman BM, Berntsen DA, Bailey MD, Zadnik K. Validation of optical coherence tomography-based crystalline lens thickness measurements in children. Optom Vis Sci. 2009; 86: 181–187. 19182701 10.1097/OPX.0b013e318198198d PMC2814774

[23] Liu S, Li H, Dorairaj S, Cheung CYL, Rousso J, Liebmann J. et al Assessment of scleral spur visibility with anterior segment optical coherence tomography. J Glaucoma. 2010; 19:132–135. 19528823 10.1097/IJG.0b013e3181a98ce4

[24] Bland J.M., Altman D.G. Statistical methods for assessing agreement between two methods of clinical measurement. Lancet. 1986; 1, 307–310. 2868172

[25] Royal College of Ophthalmologist. Guide for intravitreal injections procedure. 2009. Available:https://www.rcophth.ac.uk/wp-content/uploads/2015/01/2009-SCI-012_Guidelines_for_Intravitreal_Injections_Procedure_1.pdf. Accessed 18 June 2015.

[26] Hollands H, Wong J, Bruen R, Campbell RJ, Sharma S, Gale J. Short-term intraocular pressure changes after intravitreal injection of bevacizumab. Can J Ophthalmol. 2007; 42, 807–811. 18026202 10.3129/i07-172

[27] Boubriak OA, Urban JP, Bron AJ. Differential effects of aging on transport properties of anterior and posterior human sclera. Exp Eye Res. 2003; 76: 701–713. 12742353 10.1016/s0014-4835(03)00053-8

[28] Miao H, Wu BD, Tao Y, Li XX. Diffusion of macromolecules through sclera. Acta Ophthalmologica. 2013; 91: 1–6. 10.1111/j.1755-3768.2012.02557.x 22998133

[29] Ohno-Matsui K, Akiba M, Modegi T, Tomita M, Ishibashi T, Tokoro T. et al Association between shape of sclera and myopic retinochoroidal lesions in patients with pathologic myopia. Invest Ophthalmol Vis Sci. 2012; 7:6046–6061. 10.1167/iovs.12-10161 22879412

[30] Pekel G, Yağc R, Acer S, Ongun GT, Çetin EN, Simavl H. Comparison of Corneal Layers and Anterior Sclera in Emmetropic and Myopic Eyes. Cornea. 2015; 34:786–90 25811725 10.1097/ICO.0000000000000422

